# Microablative Erbium: YAG Laser Therapy for Vulvodynia – A Report on Efficacy, Safety, and Treatment Satisfaction

**DOI:** 10.1016/j.esxm.2021.100432

**Published:** 2021-09-20

**Authors:** Gerda Trutnovsky, Brigitte Bliem, Elfriede Greimel, Karl Tamussino, Daniela Gold

**Affiliations:** Department of Obstetrics and Gynecology, Medical University of Graz, Austria

**Keywords:** Vulvodynia, Dyspareunia, Microablative LASER, Multidisciplinary Treatment, Treatment Satisfaction

## Abstract

**Introduction:**

Treatment for vulvodynia is challenging and a multidisciplinary approach is recommended.

**Aim:**

To examine the effectiveness, safety and treatment satisfaction of vulvovaginal microablative laser treatment for vulvodynia.

**Methods:**

Case study of women who received laser treatment as part of a multidisciplinary treatment program for vulvodynia. Subjective improvement was compared to a retrospective cohort of women treated for vulvodynia without LASER therapy. LASER treatment was offered to women with vulvodynia presenting to a gynecologic pain clinic of a tertiary university hospital. LASER treatments were performed with a microablative 2,940 nm Er:YAG LASER and potentially repeated after 1 month.

**Main outcome measures:**

Change in local vulvar pain was assessed with cotton-swab tests and rated on a numeric rating scale (NRS). Treatment discomfort and short-term adverse events were recorded. The Freiburg Index of Patient Satisfaction was used to assess treatment satisfaction. Subjective symptom improvement was assessed with the Patient Global Impression of Improvement questionnaire.

**Results:**

35 women received at least 1 laser treatment, with overall mild treatment adverse effects (mean pain NRS 2.4 ± 1.9) and good treatment satisfaction (mean total score of 27.6 ±5.1; potential range 8–32). One month after last LASER treatment the pain NRS on vulvar cotton swab test improved from 6.1 ± 2.6 at baseline to 3.1 ± 2.6 (*P* < .001), and 74% of women (n = 26) reported symptom improvement. At 9–12 months follow-up 66% reported ongoing symptom improvement, with no significant difference to the control group of 32 women.

**Conclusion:**

Microablative Er:YAG vulvovaginal LASER therapy appears safe and well accepted among vulvodynia patients, but there was no significant difference in symptom improvement compared to a control group.

**Trutnovsky G, Bliem B, Greimel E, et al. Microablative Erbium: YAG Laser Therapy for Vulvodynia – A Report on Efficacy, Safety, and Treatment Satisfaction. Sex Med 2021;9:100432.**

## INTRODUCTION

Vulvodynia, defined as chronic vulvar pain without an obvious identifiable cause, is a common condition with estimated prevalence rates between 6 and 28%.^1^ According to the 2015 Consensus Terminology and Classification of Persistent Vulvar Pain vulvodynia can be described as localized or generalized, as provoked or spontaneous, or as mixed.

Pain may have been present since the first episode of vaginal penetration (ie, primary vulvodynia) or may have developed after a pain-free period (ie, secondary vulvodynia).^2^ Treatment is challenging and seems to be most successful when a multidisciplinary therapeutic approach is taken. Treatment modalities include local antinociceptive, anti-inflammatory or hormonal medications, antidepressant or anticonvulsant drugs, pelvic floor therapy, psychological interventions and vestibulectomy.^3^

In the last few years microablative and non-ablative LASER systems have been studied as conservative treatment options for the genitourinary syndrome of menopause (GSM). Some histologic studies of vaginal biopsy specimens before and after LASER exposure describe signs of neocollagenogenesis and elastogenesis, reduction of epithelial degeneration and atrophy, and an increase of fibroblasts.^4^ Clinical studies have shown that Er:Yag and CO_2_ LASER treatments may improve vaginal atrophy, GSM associated pain und dyspareunia.^5^ However, due to insufficient data the use of vulvovaginal laser therapies is controversial, and the need for further clinical trials on efficacy and safety has been emphasized.^6^

The few data on the use of LASER therapy in women with vulvodynia suggest that it may be a treatment option.^7^^,^^8^ We conducted a study of vulvar and intravaginal microablative LASER treatment for women with vulvodynia and assessed adverse effects, LASER treatment satisfaction and short and long-term efficacy.

## MATERIAL AND METHODS

The study was conducted at a gynecologic pain clinic of a tertiary university hospital that offers multidisciplinary outpatient treatment to women with vulvodynia, chronic pelvic pain and/or bladder pain syndrome. Standard assessment included a comprehensive physician led interview about presenting symptoms, gastrointestinal, urological, psychiatric and musculoskeletal comorbidities, sexual health, previous and current therapies and a full obstetric and gynecologic history. A comprehensive gynecological examination, including a thorough inspection of the vulvar skin, a cotton-swab test,^3^ myofascial trigger-point assessment and –if tolerated- a transvaginal ultrasound, was performed. Microscopy (wet prep) and ph testing of vaginal discharge was performed to exclude vulvovaginitis. A numeric rating scale (NRS), ranging from 0 “none at all” to 10 “worst imaginable,” was used for the assessment of mean pain perception at 4 defined points on the vulvar introitus during cotton-swab test. Multidisciplinary treatment involved a range of topical or oral medical treatments, pelvic floor physical therapy and psychological assessment and treatment and was offered to all patients.

Between February 2017 and April 2019 microablative LASER treatment was available to women with vulvodynia as an additional treatment option. Vulvodynia was defined according to the current Consensus Classification,^2^ and specific subtypes and associated factors were recorded. Women wishing to undergo LASER therapy received 1 to 3 treatments free of charge in an outpatient setting.

LASER treatments were performed with an 2940 nm Er:YAG Laser (Juliet, MCL31 Dermablate, Asclepion LASER Technology GmbH, Jena, Germany) according to manufacturer´s guidelines and recommendations. After application of an anesthetic cream (Emla**)** the vulva was treated with the handpiece “microspot,” which delivers a fractionated LASER beam on an area of 13 × 13 mm. All sensitive areas of the vulva were treated in an overlapping way with 2 to 3 repetitions. The pulse length was set at 300 µs and fluence that is, LASER energy delivered per unit area was modified between 15 and 30 J/cm^2^ according to the level of vulvar atrophy and patient´s acceptance. In women with vaginal atrophy the vaginal walls were included in the treatment. A vaginal probe was inserted and slowly retracted in a rotating way, resulting in a circular treatment of the entire vagina. Fluence was set at 20 J/cm^2^ (cold mode) during the first pass and at 9 J/cm^2^ (warm mode) during the second pass. At the end of treatment the level of discomfort during laser therapy was assessed with a NRS, ranging from 0 to 10. All patients had already received multidisciplinary treatment for at least 3 months. Some therapies, that is, psychological counselling, were ongoing, but no new treatment modality was started during LASER therapy.

Follow-up appointments with cotton-swab testing of vulvar pain were scheduled after 1 month and subsequently according to patients´ needs. Repeat LASER therapy was offered after a minimum of 4 weeks, depending on preliminary effectiveness and patient preference. The Freiburg Index of Patient Satisfaction (ZUF-8) was used to assess patient satisfaction with LASER treatment, with higher scores indicating higher patient satisfaction.^9^ The Patient Global Impression of Improvement (PGI-I) questionnaire was used to rate patients’ subjective symptom improvement on a 7 step Likert scale.^10^

Long term changes (9 to 12 months) in vulvar pain were assessed during follow-up visits or telephone interviews, and compared to a cohort of women who received multidisciplinary therapy for vulvodynia without LASER therapy (control group). These women did not receive LASER therapy, because they were not interested, or because they were treated before the Er:Yag LASER was available at the clinic.

In this case study all available patients with LASER treatment were included. Differences between groups were analyzed using Chi square tests and Kruskal Wallis test for categorical outcomes and paired or unpaired *t*-tests for continuous outcomes as appropriate. Pearson's correlations were used for comparisons of questionnaire results. The significance level was set to 5%. Analyses were performed using SPSS version 23 (IBM Corp, Armonk, NY, USA). The study was approved by the institutional Ethics Committee, and written informed consent was obtained from all participants.

## RESULTS

During the study period 35 women with vulvodynia were treated with Er:YAG LASER. 18 women (51%) received 1 LASER treatment, 11 women (31%) 2 treatments and 6 women (17%) 3 treatments, with a mean interval of 51 ± 27 days between treatments. The mean level of reported discomfort/pain during treatment was 2.4 ± 1.9, on a NRS ranging from 0 to 10.

At baseline, before the first LASER treatment, the cotton swab test revealed a mean pain NRS score of 6.1 (SD 2.6), which significantly improved to 3.1 ± 2.6 at the 1-month-follow-up examination after the last treatment (*P* = .001). At this time point, 26 women reported strong (34%), moderate (23%) or mild (17%) improvement. Nine women reported no change (17%) or mild deterioration (9%). Five women (14%) reported mild erythema and local swelling for up to 1 week after treatment. No severe adverse events were recorded.

At long term follow-up (9–12 months after last treatment) 12 women (34%) judged the beneficial effect of LASER treatment as “ongoing” and 15 women as temporary, with an estimated duration between “up to 2 months” (26%) and “up to 6 months” (17%).

The results of the questionnaire ZUF-8 (n = 34) showed overall high treatment satisfaction with LASER therapy, with a mean total score of 27.0 (SD 5.0; potential range 8–32). 74% of women reported that they would undergo the same treatment again if they would need it, and 94% would recommend the treatment to a friend with the same complaints. There was a significant correlation (*P* = .003) between subjective improvement (PGI-I) and patient satisfaction with LASER treatment (ZUF-8).

Subjective symptom improvement (PGI-I) was compared to a cohort of 32 women with vulvodynia, who had consecutively presented to the pain clinic, and received multidisciplinary therapy without LASER treatment. Patient characteristics, comorbidities and types of multidisciplinary therapies did not significantly differ between groups (Table 1). In the LASER group 26% reported to be “a lot better,” 17% to be “better,” 23% to be “a little better,” and 34% to be “unchanged.” In comparison, in the control group 13% reported to be “a lot better,” 41% to be “better,” 28% to be “a little better,” and 19% to be “unchanged.” Overall 73% of patients reported to be better with no significant difference in improvement between the 2 treatment groups (*P* = .6).

**Table 1:** Patient characteristics, comorbidities, and multidisciplinary therapies

	Laser group n = 35	Control group n = 32	Total n = 67	*P* value
Age (y), mean ±SD	39.7 (14.4)	44.9 (18.1)	42.2 (16.3)	.20
Menopause status				.77
premenopausal	63%	59%	61%	
postmenopausal	37%	41%	39%	
Type of vulvodynia				.87
Primary	17%	16%	18%	
Secondary	83%	84%	82%	
Provoked	91%	88%	90%	
Spontaneous	9%	12%	10%	
Comorbidities				
Pelvic floor myalgia	51%	59%	55%	.52
Recurrent vulvovaginal infections	49%	41%	45%	.52
Bladder symptoms*	43%	38%	40%	.66
Chronic pelvic pain	34%	39%	31%	.59
Psychiatric condition†	26%	25%	25%	.95
Multimodal treatment				
Psychologic counselling	57%	50%	54%	.001
Pelvic floor therapy	71%	72%	72%	.97
Topical therapies‡	80%	90%	85%	.23
Complementary therapies§	9%	16%	12%	.38

⁎ Bladder pain syndrome, urinary incontinence, overactive bladder.

† Depression, panic disorder.

‡ Fatty ointments, lidocaine, estrogen cream, local antihistamines.

§ Osteopathy, acupuncture, medical hypnosis.

## DISCUSSION

This case study of microablative Erbium:Yag LASER treatment in women with vulvodynia is the first to assess LASER therapy as part of a multidisciplinary pain treatment program, its short- and long-term efficacy and patient satisfaction. One month after last LASER treatment 74% of women reported mild, moderate or strong improvement and the mean pain scores on cotton-swab test improved from NRS 6.1 to NRS 3.1. LASER treatments were generally well tolerated with few side effects, except for 3 women who reported temporary slight deterioration of pain. Overall patient treatment satisfaction was high, even in patients with little or no improvement. However, there was no significant difference observed in subjective symptom improvement between the LASER group and a historical control group.

Similar improvement rates were found in previous studies of LASER therapy for vulvodynia.^7^^,^^8^ In a small retrospective survey 68% of women reported less pain with sexual intercourse after KTP-Nd:YAG LASER treatment.^8^ A prospective study of fractional CO2 LASER therapy found significant improvements in dyspareunia and pain scores after 3 treatment sessions.^7^

In postmenopausal women vulvar atrophy (GSM) may be one of several factors contributing to the symptoms of vulvodynia.^1^ Therefore, the positive effect of microablative LASER therapy may be explained by improvement in vascularization and increase in vaginal epithelial thickness.^4^ This finding is in accordance with a systematic review of 14 studies involving 542 postmenopausal patients with symptoms of GSM. The meta-analysis showed that LASER therapy may restore the vaginal mucosa and significantly improve symptoms of dryness and dyspareunia.^5^ In premenopausal women the mechanism of action of LASER treatment is less clear. Apart from potential tissue remodeling ^4^ and further pathophysiological mechanisms, a placebo effect is likely. A marked placebo effect of LASER therapy was seen in a randomized, placebo-controlled trial of low-level infrared LASER treatment.^11^ Thirty-four patients with provoked vestibulodynia received active or sham treatment twice weekly for 6 weeks. At study completion 78% of women receiving active LASER therapy reported improvement, compared to 44% in the placebo group. Similar strong placebo effects have been found for a range of different drug treatments for vulvodynia.^12^

Our study results are limited by the lack of a standardized treatment protocol. Women received between 1 and 3 vulvar or vulvovaginal LASER treatments according to initial response and their preference. Due to the heterogeneity of the study and the control group comparisons on effectiveness of treatment were limited. LASER treatment was offered as part of a multidisciplinary treatment program including a range of topical therapies, pelvic floor therapy and psychological counselling. These therapies are likely to be responsible for substantial symptom improvement in both study groups.

There is some evidence that about one third of vulvodynia patients will experience no relevant benefit from LASER treatment.^7^ This is not surprising considering that vulvodynia is not a specific entity but a multifactorial condition.^2^ To date no single treatment modality or uniform standard treatment has been established. Several pathophysiological mechanisms may be involved in pain development and maintenance and need to be addressed in a personalized multidisciplinary treatment program.^3^

## CONCLUSIONS

Microablative vulvovaginal LASER therapy appears safe and well accepted among vulvodynia patients, but currently there is not sufficient evidence of efficacy. Randomized placebo-controlled studies are needed to study long-term effects and identify patient groups most likely to benefit from treatment.

## STATEMENT OF AUTHORSHIP

Gerda Trutnovsky: Conceptualization, Investigation, Writing – Original Draft; Brigitte Bliem: Investigation, Statistics, Writing – Review & Editing; Elfriede Greimel: Conceptualization, Writing – Review & Editing; Karl Tamussino: Conceptualization, Writing – Review & Editing; Daniela Gold: Conceptualization, Investigation, Writing – Review & Editing.
